# The Influence of a Single Nucleotide Polymorphism within *CNDP1* on Susceptibility to Diabetic Nephropathy in Japanese Women with Type 2 Diabetes

**DOI:** 10.1371/journal.pone.0054064

**Published:** 2013-01-16

**Authors:** Mahiro Kurashige, Minako Imamura, Shin-ichi Araki, Daisuke Suzuki, Tetsuya Babazono, Takashi Uzu, Tomoya Umezono, Masao Toyoda, Koichi Kawai, Masahito Imanishi, Kazushige Hanaoka, Hiroshi Maegawa, Yasuko Uchigata, Tatsuo Hosoya, Shiro Maeda

**Affiliations:** 1 Laboratory for Endocrinology and Metabolism, RIKEN Center for Genomic Medicine, Yokohama, Kanagawa, Japan; 2 Division of Kidney and Hypertension, Department of Internal Medicine, The Jikei University School of Medicine, Tokyo, Japan; 3 Department of Medicine, Shiga University of Medical Science, Otsu, Shiga, Japan; 4 Division of Nephrology and Metabolism, Department of Internal Medicine, Tokai University School of Medicine, Isehara, Kanagawa, Japan; 5 Diabetes Center, Tokyo Women’s Medical University, Tokyo, Japan; 6 Kawai Clinic, Tsukuba, Ibaraki, Japan; 7 Department of Internal Medicine, Osaka City General Hospital, Osaka, Osaka, Japan; Kunming Institute of Zoology, Chinese Academy of Sciences, China

## Abstract

**Background:**

Several linkage analyses have mapped a susceptibility locus for diabetic nephropathy to chromosome 18q22–23, and polymorphisms within the carnosine dipeptidase 1 gene (*CNDP1)*, located on 18q22.3, have been shown to be associated with diabetic nephropathy in European subjects with type 2 diabetes. However, the association of this locus with diabetic nephropathy has not been evaluated in the Japanese population. In this study, we examined the association of polymorphisms within the *CNDP1/CNDP 2* locus with diabetic nephropathy in Japanese subjects with type 2 diabetes.

**Methodology/Principal Findings:**

We genotyped a leucine repeat polymorphism (D18S880) that is within *CNDP1* along with 29 single nucleotide polymorphisms (SNPs) in the *CNDP1*/*CNDP2* locus for 2,740 Japanese subjects with type 2 diabetes (1,205 nephropathy cases with overt nephropathy or with end-stage renal disease [ESRD], and 1,535 controls with normoalbuminuria). The association of each polymorphism with diabetic nephropathy was analysed by performing logistic regression analysis. We did not observe any association between D18S880 and diabetic nephropathy in Japanese subjects with type 2 diabetes. None of the 29 SNPs within the *CNDP1/CNDP2* locus were associated with diabetic nephropathy, but a subsequent sex-stratified analysis revealed that 1 SNP in *CNDP1* was nominally associated with diabetic nephropathy in women (rs12604675-A; *p* = 0.005, odds ratio [OR] = 1.76, 95% confidence interval [CI], 1.19−2.61). Rs12604675 was associated with overt proteinuria (*p* = 0.002, OR = 2.18, 95% CI, 1.32−3.60), but not with ESRD in Japanese women with type 2 diabetes.

**Conclusions/Significance:**

Rs12604675-A in *CNDP1* may confer susceptibility to overt proteinuria in Japanese women with type 2 diabetes.

## Introduction

Diabetic nephropathy is one of the most severe complications of diabetes mellitus, and is a leading cause of end-stage renal disease (ESRD) in both Western countries [1] and Japan [2]. Although the precise mechanisms leading to diabetic nephropathy have not yet been elucidated, genetic susceptibility is considered to be an important factor for the development and/or progression of diabetic nephropathy [3]. To date, many candidate genes for diabetic nephropathy have been evaluated, but most studies have produced conflicting results; thus, the majority of diabetic nephropathy-susceptibility genes remain to be identified.

A family-based linkage study in Turkish families suggested that a major susceptibility locus for diabetic nephropathy was linked to chromosome 18q22.3–q23 [4]. In a further study of this locus, a leucine repeat polymorphism (D18S880) in the second exon of the carnosine dipeptidase 1 gene (*CNDP1)* was shown to be significantly associated with diabetic nephropathy in a relatively small number of multi-ethnic individuals (European, Arabs, and others) with type 1 or type 2 diabetes [5]. *CNDP1* encodes a member of the dipeptidase family, which hydrolyses the substrate l-carnosine (β-alanyl-l-histidine). Carnosine is found in high concentrations in skeletal muscle and brain, and is thought to have antioxidant effects [6], anti-advanced glycation end products (AGE) effects [7], and anti-angiotensin converting enzyme (ACE) effects [8], along with a protective effect against podocyte injury [9]. Therefore, *CNDP1* is considered to be a plausible susceptibility gene candidate for diabetic nephropathy in this locus.

In the original report, homozygosity of a D18S880 allele containing 5 leucine repeats (5L), which is the most common allele found in Europeans, was found to be associated with a low frequency of diabetic nephropathy [5]. This association was also observed in European American subjects with type 2 diabetes [10], whereas other studies using Caucasian patients with type 1 diabetes [11], or African Americans [12] with type 2 diabetes, failed to observe the association of D18S880 with diabetic nephropathy. In Caucasian subjects with type 2 diabetes, Mooyaart and colleagues showed that the effect of the D18S880 was observed only in women [13]. Therefore the association of D18S880 with diabetic nephropathy remains controversial, and contribution of this locus to diabetic nephropathy has not been evaluated in Japanese subjects.

In this study, we examined the association of polymorphisms within the *CNDP1*/*CNDP2* locus, D18S880 and 29 single nucleotide polymorphisms (SNPs), with diabetic nephropathy in Japanese subjects with type 2 diabetes.

## Materials and Methods

### Subjects and DNA Preparations

DNA samples were obtained from the peripheral blood of patients with type 2 diabetes, who regularly visited the outpatient clinics at Shiga University of Medical Science, Tokai University School of Medicine, Tokyo Women’s Medical University, Osaka City General Hospital, Kawasaki Medical School, Iwate Medical University School of Medicine, Nagahama City Hospital, Daini Okamoto General Hospital, and Kawai Clinic, or were obtained from patients with type 2 diabetes registered at BioBank Japan. Diabetes was diagnosed according to the criteria of the World Health Organization. Type 2 diabetes was clinically defined as a disease with a gradual adult onset. Subjects who tested positive for anti-glutamic acid decarboxylase (GAD) antibodies and those diagnosed with mitochondrial disease (mitochondrial myopathy, encephalopathy, lactic acidosis, and stroke-like episodes [MELAS]) or maturity-onset diabetes of the young (MODY) were not included.

We conducted a case-control study involving 2,740 patients with type 2 diabetes, including 1,205 patients with overt nephropathy, as indicated by urinary albumin excretion rates (AERs) of ≥200 µg/min or urinary albumin-to-creatinine ratios (Alb/Cr) of ≥300 mg/g Cr, or with end stage renal disease (ESRD), and 1,535 controls with normoalbuminuria indicated by AERs of <20 µg/min or Alb/Cr of <30 mg/g Cr, and diabetic retinopathy, or with normoalbuminuria and a long duration of diabetes (>10 years). AER or ACR were measured at least twice for each patient. The characteristics of the study participants are listed in Table 1.

**Table 1:** Clinical characteristics of the participants.

	Nephropathy case	Control	p-value
N	1,205	1,535	
Sex (M:F)	807∶ 394^a^	887∶ 634^b^	<0.001
Age (years)#	62.1±11.3	65.3±9.8	<0.001
BMI (kg/m^2^)#	23.3±3.8	23.6±3.6	NS
HbA1c (%)#	7.5±3.2	7.8±1.2	0.06
SBP (mmHg)#	140±20	131±17	0.001
DBP (mmHg)#	75±12	74±11	NS
Diabetes duration (years)#	18.1±12.1	15.4±7.9	<0.001

Abbreviations: BMI, body mass index; HbA1c, hemoglobin A1c; SBP, systolic blood pressure; DBP, diastolic blood pressure.

a 4 unknown,

b 14 unknown.

# Mean ± SD, HbA1c; NGSP.

### Ethics Statements

All the participants agreed to the protocol of this study and provided written informed consent. The study protocol conformed to the provisions of the Declaration of Helsinki, and was approved by the ethics committees of RIKEN Yokohama Institute and each of the participating institutes, Shiga University of Medical Science, Tokai University School of Medicine, Tokyo Women’s Medical University, Osaka City General Hospital, Kawasaki Medical School, Iwate Medical University School of Medicine, Nagahama City Hospital, Daini Okamoto General Hospital, and Toride Kyodo Hospital, to which the Kawai Clinic is affiliated.

### Genotyping of D18S880

D18S880, in exon 2 of *CNDP1,* was genotyped by performing a fragment length analysis on an ABI 3500×L Genetic Analyzer (Life Technologies Corporation, Carlsbad, CA). The primers used were; forward, 5′-[6-FAM]-AGG CAG CTG TGT GAG GTA AC-3′, reverse, 5′-GGG TGA GGA GAA CAT GCC-3′. Polymerase chain reaction (PCR) product lengths of 168 bp and 165 bp, respectively, were confirmed as fragments with 6 leucine repeats and 5 leucine repeats, respectively by direct sequencing. In each assay, the genotype of each product was compared to that of the control fragments containing 5 CTG repeats, in addition to a size standard marker. Fragment length was determined using the GeneMapper® software v3.7 (Life Technologies Corporation, Carlsbad, CA).

### SNP Genotyping

The HapMap database (http://hapmap.ncbi.nlm.nih.gov/) was searched for SNPs within the *CNDP1*/*CNDP2* locus, and 25 tagging SNPs were selected: rs11661606, rs11665154, rs11876996, rs12326826, rs12327522, rs12605520, rs12957330, rs12964454, rs17089368, rs17089390, rs17817077, rs2241508, rs2346064, rs4329999, rs4892239, rs12604675, rs17817095, rs6566815, rs7229005, rs7239132, rs7244647, rs733686, rs747174, rs8087768, and rs9953129. We also selected additional 4 SNPs, which were examined in a previous report [14] and not included in the above tagging SNPs: rs12456388, rs2346061, rs4891558, and rs7244370. Each SNP genotype was analyzed using a multiplex PCR-invader assay, as previously described [15]. Genotyping success rates for all SNPs were over 95%, and concordance rates in 96 randomly selected duplicated samples were 100%.

### Statistical Analysis

Descriptive analyses of characteristics data were performed using t-tests. Genotype distributions for Hardy-Weinberg equilibrium (HWE) proportions were tested using a χ^2^ test. The differences between the case and control groups in terms of genotype distribution scored with an additive model were evaluated using a logistic regression analysis, adjusting for sex, age, log-transformed body mass index (BMI), and duration of diabetes.

The level of significance was determined by the Bonferroni’s method for correcting multiple testing errors. Under the selected 30 variants and 4 subgroup analyses (sex stratified [men and women] and phenotype stratified [proteinuria and ESRD]), a *p* value of less than 0.0004 (0.05 divided by [30×4]) was considered statistically significant.

## Results

We first examined the association of D18S880 with diabetic nephropathy. As shown in Table 2, subjects with the 6L–6L genotype were most common (94.3%), followed by subjects with the 6L–5L genotype (5.5%), while other genotypes were very rare (<0.1%) in the analyzed Japanese population. There were significant differences in the genotype distribution of D18S880 between Japanese and other ethnic groups, such as Europeans [5], [10], [14], South Asians [16], and African Americans [12]. We did not observe a significant association of D18S880 with diabetic nephropathy (χ^2^ test, *p* = 0.58) in Japanese subjects with type 2 diabetes.

**Table 2:** Association of D18S880 with diabetic nephropathy.

	Number of patients (%)				
genotype	6L–6L	6–5	5–5	6–4	6–8
Nephropathy cases	1128 (94.7)	62 (5.2)	1 (0.1)	0	0
Controls	1429 (94.0)	88 (5.8)	1 (0.07)	2 (0.1)	1 (0.07)
	NS^a^				
European^c^	(10.8)	(47.0)	(41.3)	<1.0	–
South Asian^c^	(31.4)	(40.3)	(27.2)	(1.0)	–
African American^d^			(39.0)^b^		

a chi-square test.

b 4L/5L and 5L/5L.

c Genotype frequencies in general population for Europeans and South Asians reported by Mooyaart et al. (2009) Diabetes Res and Clin Prac 85∶272–278.

d Data in healthy controls for African Americans reported by McDonough et al. (2009) Hum Genet 126∶265–275.

We next examined the association of 29 SNPs within the *CNDP1*/*CNDP2* locus with diabetic nephropathy. The genotype distributions of all SNPs did not deviate from HWE proportions, except for rs17089368 in the control (*p* for HWE test = 0.013), and rs9953129 in nephropathy case (*p* for HWE test = 0.035) (Table S1).

**Figure 1:**
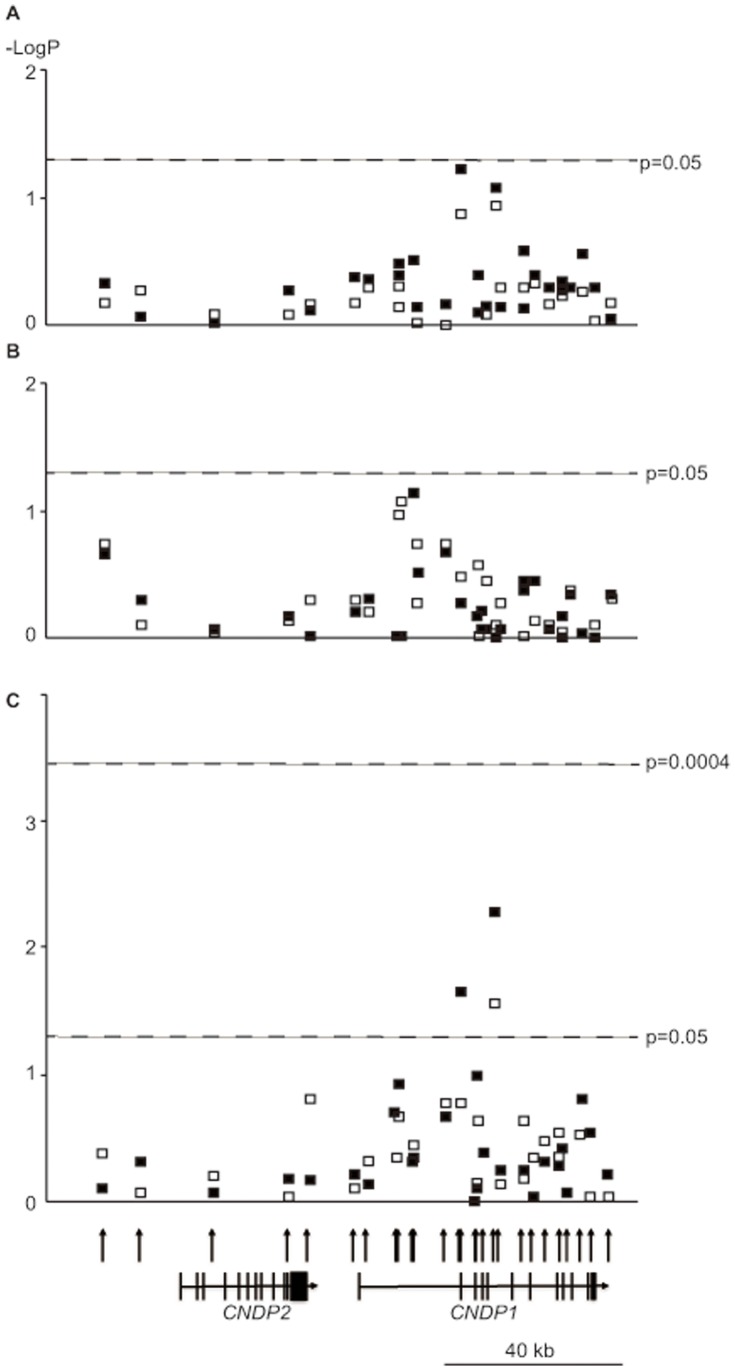
Association of single nucleotide polymorphisms (SNPs) within *CNDP1/CNDP2* locus with diabetic nephropathy. Results of association studies are shown using: A) men and women, B) men only, or C) women only. The x-axis represents the position in chromosome 18, and the y-axis shows the absolute values of log_10_-transformed association *p* values. Open squares represent unadjusted values, and black squares represent values adjusted for age, log-transformed body mass index, and duration of diabetes. Thresholds for nominal (*p* = 0.05) or statistical (*p* = 0.0004) significance are shown as broken lines.

None of the 29 SNPs showed a significant effect on susceptibility to diabetic nephropathy (*p*≥0.05, Figure 1A, Table S2, S3, S4, S5). Subsequent sex-stratified analysis, however, revealed that 2 SNPs were nominally associated with diabetic nephropathy in women (rs12604675-A: *p* = 0.005, OR = 1.760, 95% CI, 1.186–2.614, and rs733686-T: *p* = 0.022, OR = 1.333, 95% CI, 1.043–1.704, Figure 1C, Table S2). Interestingly, rs12604675-A was observed to be associated with overt proteinuria (*p* = 0.002, OR = 2.178, 95% CI, 1.318–3.600, Table 3, Figure S1), but not associated with ESRD in Japanese women with type 2 diabetes. No haplotype within this locus had a stronger association with diabetic nephropathy than rs12604675 alone in the present population (Table S6).

**Table 3:** Association of rs12604675-A with diabetic nephropathy.

Nephropathy	p-value	OR	95%Cl
All	0.0813	1.256	0.972−1.623
Overt proteinuria	0.3738	1.173	0.825−1.668
ESRD	0.0997	1.289	0.953−1.745
Men	0.9749	0.995	0.709−1.396
Overt proteinuria	0.1863	0.707	0.423−1.182
ESRD	0.3433	1.203	0.821−1.760
Women	0.005	1.760	1.186−2.614
Overt proteinuria	0.0024	2.178	1.318−3.600
ESRD	0.1371	1.459	0.887−2.401

OR represents the odds ratio per copy of risk allele (A). P-values were calculated using a logistic regression analysis with additive model (adjusted for sex, age, log-transformed body mass index and duration of diabetes).

## Discussion

In this study, we examined the association of polymorphisms within the *CNDP1*/*CNDP2* locus with diabetic nephropathy in Japanese patients with type 2 diabetes, and found that a SNP in intron 5 of the *CNDP1*, rs12604675 (Figure S2), was nominally associated with overt proteinuria in Japanese women with type 2 diabetes.

Results from several linkage analyses suggested the existence of a susceptibility locus to diabetic nephropathy on chromosome 18q22.3–q23 [4], [17], [18]. *CNDP1*, located at 18q22.3, encodes carnosinase 1, which is found in human plasma and brain and degrades carnosine, homocarnosine [19]. Carnosine has been reported to protect against renal injury, along with having antioxidant activity [6], inhibition of AGE formation [7], and ACE inhibition property [8], suggesting that *CNDP1* contributes to conferring susceptibility to diabetic nephropathy.

A trinucleotide repeat (leucine repeat) polymorphism, D18S880, in exon 2 of *CNDP1* was previously reported to be associated with diabetic nephropathy. Patients with homozygous alleles containing 5 leucine repeats were shown to be less susceptible to diabetic nephropathy, and to have lower serum carnosinase activities as compared to individuals with other genotypes [5]. It was also shown in COS cells that CNDP1 secretion was significantly higher in the cells expressing *CNDP1* variants with more than 5 leucines [20]. However, in the present Japanese population, we did not observe any association of this leucine repeat polymorphism with diabetic nephropathy. There were remarkable differences in the genotype frequencies of D18S880 between the Japanese population and other ethnic groups, with the protective 5L–5L genotype being the most common (23–41%), in European [5], [10], [14], South Asian [16], or African American populations [12], whereas individuals with the 5L-5L genotype were very rare in the present Japanese population (<0.1%). The results of our genotyping for D18S880 were validated by direct sequencing of fragments corresponding to either the 5L or 6L allele, and validated using a control genotype from genomic DNA extracted from human embryonic kidney (HEK293) cells, derived from a Caucasian individual identified as 5L-5L in our fragment analysis (data not shown). In addition, the genotype distribution of D18S880 in the present study is similar to that in Chinese peritoneal dialysis patients [21], with frequencies of 5L–5L and 6L–6L at 0.9% and 80% respectively. Therefore, differences in the genotype distribution between East Asian populations and other ethnic populations truly exists, and may be a principal cause for the discrepancy between the results of the previous study and the present Japanese study.

To further evaluate the contribution of this locus to diabetic nephropathy susceptibility, we examined the association of 29 SNPs, including 25 tagging SNPs, with diabetic nephropathy. The results indicated that 2 SNPs in *CNDP1*, rs12604675 and rs733686, were nominally associated with diabetic nephropathy only in women. These 2 SNPs were in weak LD (r^2^ = 0.04), but a conditional analysis including both SNPs using a logistic model revealed that the association of rs12604675 was still significant after conditioning (*p* = 0.0309), whereas the association of rs733686 was no longer significant after conditioning (*p* = 0.1029), indicating that these 2 SNPs were not independent, and that rs12604675 is probably more closely linked to disease susceptibility. Since, of many genes reported to be susceptible to diabetic nephropathy, some had similarly affected both proteinuria and ESRD but the others had not. Therefore, we next performed phenotype-stratified analyses, proteinuria or ESRD. Interestingly, rs12604675-A was associated with overt proteinuria, but not with ESRD in Japanese women with type 2 diabetes. Discrepancies in genomic loci underlying susceptibility to proteinuria versus ESRD have previously been noted in a genome-wide linkage study for diabetic nephropathy in type 2 diabetes [22]. As most of the patients with ESRD were considered to have had proteinuria, some selection bias, such as a survival effect, may have existed when patients with ESRD were included. Since the presence of proteinuria is also recognized as a predictor of cardiovascular diseases, the association of rs12604675 with proteinuria may reflect an association between the gene and cardiovascular diseases or metabolic syndrome instead.

Though it remains unknown why the association of *CNDP1* SNPs with diabetic nephropathy was observed only in women, there are several reports demonstrating that the effects of *CNDP1* variants are more significant in women than in men [13], [14], [23]. There are several possibilities to explain the predominant effect of *CNDP1* variants in women. First, it has been reported that there are differences in muscle carnosine content between men and women, with muscle carnosine content in men shown to be higher than that in women [24]. Since it has also been shown that carnosine content in the muscle of female mice is elevated approximately 2.5-fold after administration of testosterone [25], tissue carnosine contents have been considered to be regulated by the anabolic action of testosterone, which may mask the effect of *CNDP1* variants in men. Second, in the present population, men had a higher risk than women for diabetic nephropathy (*p* = 4.96×10^−5^, OR = 1.39, 95% CI, 1.19−1.64). Therefore, the effects of rs12604675 on conferring susceptibility to diabetic nephropathy may also be masked in men. The elucidation of a precise mechanism will require further investigation.

Ahluwalia *et al.* reported the association of 2 SNPs located in the regulatory region of *CNDP1* and *CNDP2*, rs2346061 and rs7577, with diabetic nephropathy in Swedish subjects with type 2 diabetes [14]. They also found a haplotype consisting of 3 SNPs (rs7244370 in addition to the above-mentioned 2 SNPs), which was associated with a 3-fold increased risk of diabetic nephropathy. Another study in American Indians with type 2 diabetes found nominal association of 2 SNPs within *CNDP1*, rs12957330 and rs17817077, with ESRD [26]. We analyzed these SNPs in the present study of Japanese patients with type 2 diabetes. The results indicated that rs7577 was monoallelic in the Japanese, and we also did not observe the association of either the SNPs or the haplotype (rs2346061[C]–rs7244370[G]). Since nephropathy cases in the Swedish study included patients with microalbuminuria, the discrepancy between the results of those studies and our study may be explained by phenotype differences and/or ethnic differences.

There were some potential limitations in this study. First, in this association study, we recruited participants who met the criteria of case and control as many as possible to avoid the reduction of study power, and did not perform matching procedures. As a result, there were significant differences in age, sex ratio between the nephropathy cases and controls. Although we adjusted these parameters by including them in a same logistic model in our association study, these mismatches might affect the results of our association study.

Second, sample size in the present study was not sufficiently large enough to detect smaller effect loci. The present Japanese sample has 100% power for most SNPs with OR ≥2.0, whereas, for SNPs with OR of 1.10, estimated power is less than 50% (Table S7, CaTS power calculator, CaTS: http://www.sph.umich.edu/csg/abecasis/CaTS/)].

Third, rs12604675, with suggestive evidence for an association in the present study, was not examined in Swedish or American Indian populations, and the association of rs12604675 in the present study did not overcome correction for multiple testing errors. Therefore, the association of rs12604675 needs to be further evaluated in independent samples.

In conclusion, on performing an extensive analysis for analyzing the association of the *CNDP1*/*CNDP2* locus with diabetic nephropathy in Japanese subjects with type 2 diabetes, we obtained suggestive evidence that rs12604675-A contributes to susceptibility to overt proteinuria in Japanese women with type 2 diabetes, although the conclusion in this study need to be validated in an independent cohort.

## Supporting Information

Figure S1 **Association of SNPs within CNDP1-CNDP2 locus with diabetic nephropathy in Japanese women with type 2 diabetes.** Results of association studies using A) patients with ESRD and controls B) patients with overt proteinuria and control. X-axis represents position in the chromosome 18, and y-axis shows absolute values of log10-transformed association p values. Open squares, un-adjusted, black squares, adjusted for age, log-transformated BMI and duration of diabetes. Thresholds for nominal (p = 0.05) or statistical (p = 0.0004) significance are shown as broken lines.
(PDF)

Figure S2 **Linkage disequilibrium structure for CNDP1-CNDP2 locus in the Japanese population.** Pairwise correlation structure analyzed by Haploview software (Haploview: http://www.broadinstitute.org/haploview/haploview). The plot includes pairwise D’ values from Hapmap release 27 for the JPT (Japanese in Tokyo, Japan). Each arrow indicates position of each SNP examined in this study.
(PDF)

Table S1 **Genotype and allele frequencies for polymorphisms in** ***CNDP1*** **/** ***2*** **locus in the present Japanese population.**
(XLSX)

Table S2 **Association between SNPs within** ***CNDP1*** **/** ***2*** **and diabetic nephropathy (additive model).** P-values were calculated using a logistic regression analysis with additive model (adjusted for, age, sex, log-transformed BMI and duration of diabetes) * Effect allele frequency.
(XLSX)

Table S3 **Association between SNPs within** ***CNDP1*** **/** ***2*** **and diabetic nephropathy (additive model, unadjusted).** P-values were calculated using a logistic regression analysis with additive model * Effect allele frequency.
(XLSX)

Table S4 **Association between SNPs within** ***CNDP1*** **/** ***2*** **and diabetic nephropathy (dominant model).** P-values were calculated using a logistic regression analysis with dominant model (adjusted for, age, sex, log-transformed BMI and duration of diabetes) * Effect allele frequency.
(XLSX)

Table S5 **Association between SNPs within** ***CNDP1*** **/** ***2*** **and diabetic nephropathy (recessive model).** P-values were calculated using a logistic regression analysis with recessive model (adjusted for, age, sex, log-transformed BMI and duration of diabetes) * Effect allele frequency.
(XLSX)

Table S6 **Association of haplotypes within** ***CNDP1*** **/** ***CNDP2*** **locus with diabetic nephropathy.** The analyses for haplotype structures and association study were performed using Haploview software version 4.1.
(DOCX)

Table S7 **Estimation of statistical power for the present study.** Power estimation was performed using CaTS power calculator, CaTS: http://www.sph.umich.edu/csg/abecasis/CaTS/) The prevalence of diabetic nephropathy is assumed to be 10%, α = 0.05.
(XLSX)
