# Supplementary material for: The Influence of a Single Nucleotide Polymorphism within CNDP1 on Susceptibility to Diabetic Nephropathy in Japanese Women with Type 2 Diabetes
Source: PLoS One. 2013 Jan 16;8(1):e54064. doi: 10.1371/journal.pone.0054064 (PMC3546962; doi:10.1371/journal.pone.0054064)
Supplement: Figure S1 — Association of SNPs within CNDP1-CNDP2 locus with diabetic nephropathy in Japanese women with type 2 diabetes. Results of association studies using A) patients with ESRD and controls B) patients with overt proteinuria and control. X-axis represents position in the chromosome 18, and y-axis shows absolute values of log10-transformed association p values. Open squares, un-adjusted, black squares, adjusted for age, log-transformated BMI and duration of diabetes. Thresholds for nominal (p = 0.05) or statistical (p = 0.0004) significance are shown as broken lines. (PDF) [file pone.0054064.s001.pdf]

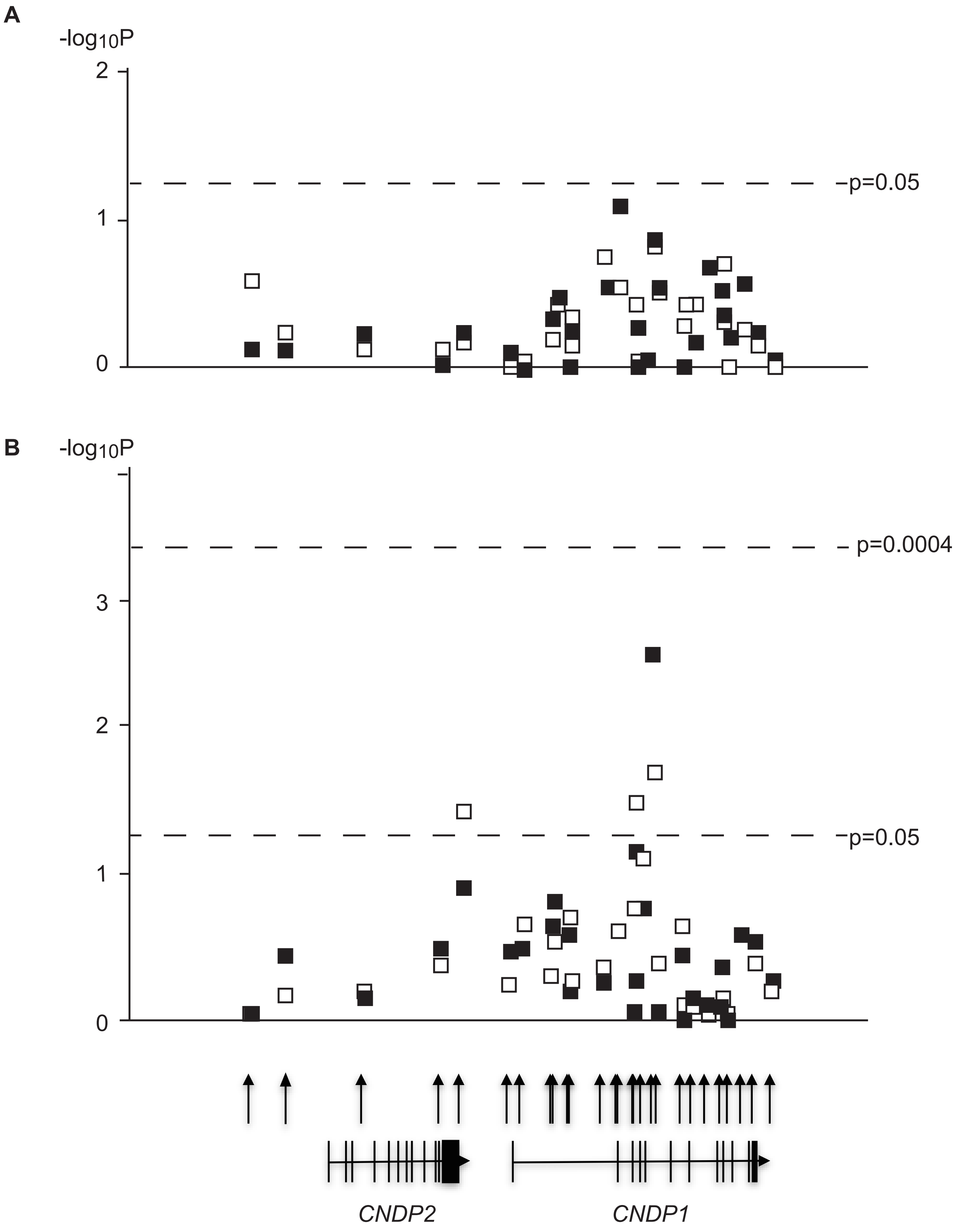

Figure S1 Association of SNPs within *CNDP1-CNDP2* locus with diabetic nephropathy in Japanese women with type 2 diabetes

Results of association studies using A) patients with ESRD and controls B) patients with overt proteinuria and control. X-axis represents position in the chromosome 18, and y-axis shows absolute values of $\log_{10}$-transformed association p values. Open squares, un-adjusted, black squares, adjusted for age, log-transformated BMI and duration of diabetes. Thresholds for nominal ($p=0.05$) or statistical ($p=0.0004$) significance are shown as broken lines.
