# Supplementary material for: The Influence of a Single Nucleotide Polymorphism within CNDP1 on Susceptibility to Diabetic Nephropathy in Japanese Women with Type 2 Diabetes
Source: PLoS One. 2013 Jan 16;8(1):e54064. doi: 10.1371/journal.pone.0054064 (PMC3546962; doi:10.1371/journal.pone.0054064)
Supplement: Figure S2 — Linkage disequilibrium structure for CNDP1-CNDP2 locus in the Japanese population. Pairwise correlation structure analyzed by Haploview software (Haploview: http://www.broadinstitute.org/haploview/haploview). The plot includes pairwise D’ values from Hapmap release 27 for the JPT (Japanese in Tokyo, Japan). Each arrow indicates position of each SNP examined in this study. (PDF) [file pone.0054064.s002.pdf]

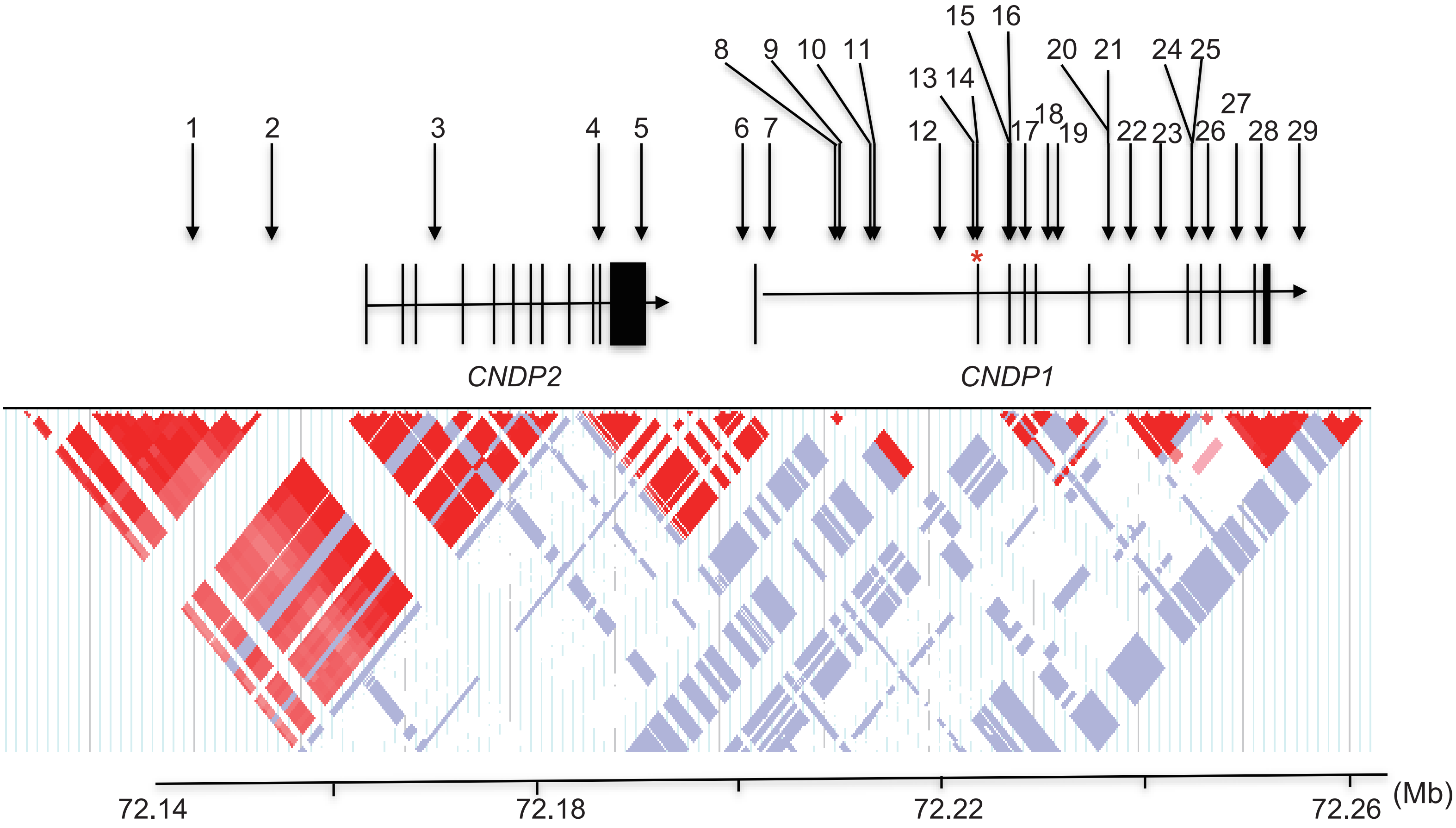

Figure S2. Linkage disequilibrium structure for CNDP1-CNDP2 locus in the Japanese population

Pairwise correlation structure analyzed by Haploview software

(Haploview: http://www.broadinstitute.org/haploview/haploview),

The plot includes pairwise D' values from Hapmap release 27 for the JPT (Japanese in Tokyo, Japan)

Each arrow indicates position of each SNP examined in this study.

(1)rs11665154 (2)rs747174 (3)rs4891558 (4)rs2241508 (5)rs17089368 (6)rs2346061 (7)rs7244370 (8)rs17817077 (9)rs17817095 (10)rs8087768 (11)rs2346064 (12)rs4892239 (13)rs733686 (14)rs12605520 (15)rs7239132 (16)rs17089390 (17)rs4329999 (18)rs12604675 (19)rs11876996 (20)rs12327522 (21)rs12326826 (22)rs12964454 (23)rs12456388 (24)rs9953129 (25)rs11661606 (26)rs7229005 (27)rs7244647 (28)rs12957330 (29)rs6566815

*D18S880
