# Supplementary material for: The Influence of a Single Nucleotide Polymorphism within CNDP1 on Susceptibility to Diabetic Nephropathy in Japanese Women with Type 2 Diabetes
Source: PLoS One. 2013 Jan 16;8(1):e54064. doi: 10.1371/journal.pone.0054064 (PMC3546962; doi:10.1371/journal.pone.0054064)
Supplement: Table S6 — Association of haplotypes within CNDP1 / CNDP2 locus with diabetic nephropathy. The analyses for haplotype structures and association study were performed using Haploview software version 4.1. (DOCX) [file pone.0054064.s008.docx]

**Table S6** Association of haplotypes within *CNDP1*/*CNDP2* locus with diabetic nephropathy

|  | Frequency | |  |
| --- | --- | --- | --- |
|  | case | control | P |
| Block 1: rs2241508, rs17089368 | | | |
| AT | 0.669 | 0.667 | 0.9194 |
| AC | 0.228 | 0.231 | 0.7512 |
| GT | 0.103 | 0.100 | 0.7131 |
| Block 2: rs2346061, rs7244370 | | | |
| AG | 0.888 | 0.872 | 0.6570 |
| CT | 0.072 | 0.069 | 0.6010 |
| CG | 0.056 | 0.036 | 0.8498 |
| Block 3: rs17817077, rs17817095 | | | |
| CT | 0.893 | 0.897 | 0.6179 |
| TC | 0.106 | 0.101 | 0.5823 |
| Block 4: rs8087768, rs2346064, rs4892239 | | | |
| TTA | 0.819 | 0.813 | 0.5986 |
| GCG | 0.093 | 0.095 | 0.7965 |
| GCA | 0.066 | 0.065 | 0.8893 |
| GTA | 0.021 | 0.024 | 0.4039 |
| Block 5: rs12605520, rs7239132, rs17089390 | | | |
| CAT | 0.518 | 0.521 | 0.7990 |
| TCT | 0.330 | 0.336 | 0.6523 |
| CCC | 0.082 | 0.081 | 0.8580 |
| CCT | 0.068 | 0.059 | 0.1745 |
| Block 6: rs12604675, rs11876996 | | | |
| GC | 0.562 | 0.565 | 0.8762 |
| GT | 0.372 | 0.380 | 0.5229 |
| AC | 0.066 | 0.055 | 0.1031 |
| Block 7: rs12964454, rs12456388, rs9953129 | | | |
| GCG | 0.534 | 0.524 | 0.4656 |
| TTC | 0.244 | 0.252 | 0.4894 |
| TCG | 0.215 | 0.217 | 0.8168 |
| Block 8: rs7244647, rs12957330, rs6566815 | | | |
| CGT | 0.650 | 0.653 | 0.8403 |
| CGG | 0.186 | 0.175 | 0.2911 |
| TGG | 0.098 | 0.106 | 0.3225 |
| TAG | 0.066 | 0.066 | 0.9696 |

The analyses for haplotype structures and association study were performed using Haploview software version 4.1.
